# Turning over New Leaves

**Published:** 1892-03-19

**Authors:** 


### Turning Over New Leaves.

The "Sunday at Home" for this month contains some very interesting articles. "Religious Life and Thought in Holland" is a brief but interesting history of the progress and development of religious thought in Holland. It is illustrated with two excellent portraits of the leaders of different periods. "Wanderings in the Holy Land," a familiar subject, is treated in an interesting manner by Adelia Gates, and is charmingly illustrated. "Tom Heron of Iat," the serial story, is a story of the Revival Times and of John Wesley. It is well told and consistent. "Immortelles" is a touching little tale of hospital interest.

The "Leisure Hour" for March abounds in matter of interest to every class of reader. The series running through its pages entitled "The Great London Dailies" is devoted this month to the "DailyNews," and Mr. Massingham has provided us with quite an absorbing little narrative. "The Statesmen of Europe" are General Vannonsky, M. de Giers, and M. Pobiedonostzeff. E. H. Hickey contributes simple but affecting memorial lines to the late Prince. There is an excellent article on "His Excellency the Viceroy Li Hung Chang." "In Spite of Herself" and "The Romance of Aunt Hilary and Uncle Higgins" are well written and interesting. The young folks' periodicals, "The Girl's Own Book" and the "Boy's Own Book" are as full of information

